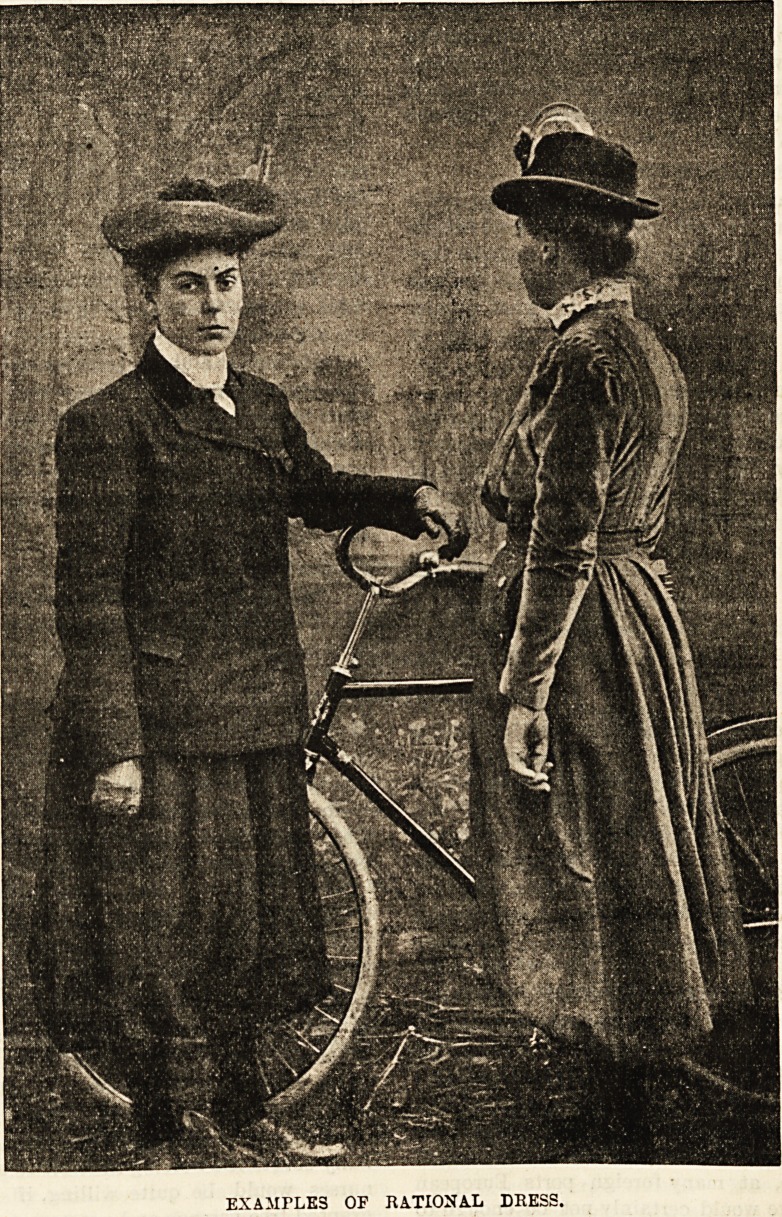# "The Hospital" Nursing Mirror

**Published:** 1901-09-07

**Authors:** 


"The Hospital" Nursing Mirror.

Being the Nursing Section of "The Hospital."

[Contributions for this Section of "The Hospital" should be addressed to the Editor, "The Hospital" Nursing Mirror, 28 & 29 Southampton Street Strand, London, W.C.]

### Notes on News from the Nursing World.

### OUR CHRISTMAS DISTRIBUTION.

Probably there are nurses who have remembered our Christmas Distribution during their holidays. But as the holiday season is not yet over, some may be glad to be reminded that every year an increasing number of patients in hospitals and infirmaries receive, through the kindness of our readers, a present of warm articles of clothing at Christmas, in order that on their discharge they may leave the institution suitably clad. No one knows better than a nurse how serviceable this form of charity is, and we only mention the subject thus early so that no opportunity of preparation may be lost.

### THE WOMEN'S MEMORIAL TO QUEEN VICTORIA.

The various Parliamentary divisions of Lincolnshire are being organised under the auspices of Lady Brownlow, for the purpose of raising funds for the Jubilee Nurses' Institute as the Women's National Memorial to Queen Victoria, and a number of meetings are being arranged. On Saturday, September 14th, there will be one at Fillingham Castle for which the president of the Gainsborough division has sent out invitations to ladies of all denominations and political opinions in the constituency. A committee will then be formed to carry out the organisation of the division. We should like to hear that presidents of other divisions are equally active, as it is most important that the movement should be in full swing at an early period of the autumn.

### THE NORTHAMPTON MEMORIAL.

We are glad to learn that Princess Christian has consented to perform the ceremony of opening the new handsome nursing institute at Northampton, which has been erected as a local memorial to Queen Victoria. The date of the interesting event has not yet been definitely fixed, but the second week in December is mentioned. The Northampton people must be congratulated on the fact hat, while some other towns have been thinking about a memorial to the late Sovereign, they have completed their scheme. The present quarters of the Nursing Association are in Hazelwood Road, and the staff comprises twenty nurses of whom five are Queen's nurses.

### THE VALUE OF COLLECTING CARDS.

It is interesting to learn that the collecting card first adopted in Ireland by Lady Clonbrook as a means of obtaining contributions to the fund for the Irish Women's Memorial to Queen Victoria, has been already widely used with signal success. Upwards of £300 has been collected by this method in Dublin, largely in small sums from the poorer classes. It has wisely been decided to keep open the subscription list in the sister island, at the instance of a lady who said that in her district nearly £100 had been collected and only five cards had yet been returned. We agree with the Countess of Fingall as to the necessity of it being emphasised that none of the money obtained in Ireland will be sent to England. The Irish Committee properly rejected a suggestion that the money collected in the different districts should be employed solely for their benefit; but whilst it is better to have a central fund, the entire amount contributed should certainly be applied to the Irish Memorial.

### THE PROGRAMME OF THE INTERNATIONAL NURSES' CONGRESS.

The International Nurses' Congress, which opens at Buffalo on Wednesday the 18th, will close on Friday the 20th, but on Saturday the 21st, which is to be known as Trained Nurses' Day, the Temple of Music in the Exposition grounds will be placed at the disposal of the delegates for a supplementary conference. The address of the president of the Congress, Miss Isabel McIsaac, superintendent of Illinois Training School for Nurses at Chicago—whose account of this institution in our columns will not have been forgotten—will be the feature of the opening session. To Miss Stewart, matron of St. Bartholomew's Hospital, and Miss Mollett, matron of the Royal South Hants Hospital, Southampton, has been entrusted the task of treating the important subject of "Hospital Administration in Great Britain." It is, of course, impossible for these ladies to present an exhaustive view of the subject. Mrs. Hampton Robb will be considered a sufficient authority on the question of "Women on Hospital Boards," from an American standpoint. In the afternoon of Wednesday, Miss Kimber will doubtless do justice to "The Nurses' Co-operation," and in present circumstances her remarks and the ensuing discussion will excite lively interest on both sides of the Atlantic. On Thursday, Mrs. Strong, the capable matron of the Royal Infirmary at Glasgow, will deal with "The Preparatory Instruction of Nurses," and Miss L. L. Dock's contribution, "What we are doing with the Three Years' Course," should contain much useful information. Subsequently there will be discussions on "Private Nursing," "District Nursing," and "Army Nursing." We are glad to see that Miss Amy Hughes is to introduce the topic of "District Nursing in England," with which she is fully acquainted; and that Miss Honnor Morten is to speak on the "London School Nurses' Society," of which she probably knows more than anyone else. Miss Arkle and Miss Watt, of the Indian Army Nursing Service, should be able to make some practical suggestions on "Army Nursing," on which Miss Eugenie Hibbard, who is to discourse on "Hospital Work in Cuba," is an expert. The last subject on the paper is "Organisation and Legislation in the Nursing Profession," in respect to which Miss C. J. Wood, formerly lady superintendent at the Great Ormond Street Hospital for Sick Children, greatly daring, promises not only a retrospect, but also a forecast.

### ANOTHER CHRISTMAS IN SOUTH AFRICA.

From time to time we record the arrival of two or three nursing sisters from South Africa, but the great majority are still kept at their posts. If, however, the apprehensions expressed by the writer of an article on "Nursing at Warmbad," in our issue of last week, are well founded, we think that the War Office should take steps to afford the relief which, in most cases, must be required. Our contributor, herself a member of the Army Nursing Service Reserve, says she is very much afraid that the nurses "will see another Christmas in South Africa." Some there may be who are physically strong enough not to be alarmed at this prospect, but a considerable number who have already been out there fully eighteen months ought certainly to have a holiday. That an ample staff of nurses is an urgent necessity is only too abundantly shown from the long array of names under the heading "Dangerously Ill" in Tuesday's papers. But this is an additional reason why those who have been working hard for weary months, and who if at all run down are more susceptible to infection, should be replaced by nurses fresh from England. There was a picture in the *Graphic* last week showing sisters playing cricket. But even an occasional game at cricket, or hockey, will not suffice to prevent nurses from breaking down under an unduly prolonged strain; and if the war is likely to drag on until the end of the year, a substantial detachment of the Army Service Reserve should be despatched forthwith to South Africa in order to enable those members who have been on duty since spring of last year to return home.

### LADY HARBERTON ON NURSES' UNIFORMS.

To our readers the most interesting part of the article which Lady Harberton contributes to our columns this week on "A Fashion Producing Disease" will be her severe strictures on the dress usually worn by nurses in the pursuit of their calling. As Lady Harberton says, there are many objections to this costume, and on purely hygienic principles the dress adopted by a vivandière—and, in a modified form, by Belgian army nurses—has much to recommend it. The insular prejudice against anything "mannish" in the pursuit of such an essentially feminine avocation as nursing will not, however, be removed for many years to come. But a step in the right direction would certainly be the initiation by the authorities of the leading civil hospitals of a rule that the uniform skirts of all nurses should be two inches from the ground. The need of some reform in the Army Nursing Service has been very clearly brought home to the sisters-in charge of camps in South Africa during the wet and rainy season, when, as one of our contributors at the front once wrote, it was a case of "simply wading ankle-deep in water"; and the War Office could not do better than introduce Lady Harberton's rational skirt for use when attendance on patients involves getting from one tent to another in every variety of weather.

### BELFAST NURSES IN A CULTURE BED OF TYPHOID.

There must be something very wrong in respect to the conditions of nursing at the Belfast Workhouse Infirmary, when nurses are constantly leaving, and eight members of the staff are down with typhoid fever. At the last meeting of the Belfast Board of Guardians an attempt was made to explain the former state of affairs on the ground that the Belfast Workhouse Infirmary is such a splendid training school that the nurses are constantly offered better appointments elsewhere. This pleasant theory, however, was discredited by several speakers, one of whom mentioned that the charge nurses, who had not left, complained very strongly of the discomforts of their position. As to the prevalence of typhoid, Dr. Ritchie made the startling allegation that the nurses in the hospital are "living in a culture bed of typhoid fever." The chairman having intimated that such a serious assertion was not warranted, Dr. Ritchie retorted that "the hospital has drains leaking and closets that cannot be emptied, whereupon the chairman was driven to admit that there might be "some grounds" for it. Meanwhile, the Board fall back on the fact that the infirmary committee, to whom the question had been referred, have recommended that "a fowl once a week" shall be provided for the delectation of the nurses recovering from typhoid, and suggested the concession of a "few additional articles" for the benefit of the staff generally. That the Belfast Guardians should imagine that these puerile recommendations will put a stop to the exodus of nurses, terminate complaints, and settle the burning sanitary question, is as extraordinary as it is reprehensible. We cannot understand how men who recognise moral responsibility can allow those in their employment to continue daily exposed to disease and death when a remedy lies ready to hand.

### AMERICAN ARMY NURSES AND DR. ANITA McGEE.

Dr. Anita Newcomb McGee, President of the Spanish-American War Nurses, who this year will hold their annual meeting at Buffalo, immediately preceding the International Congress of Nurses, has been presented by the Army Nurse Corps in the United States with one of the finest grade American watches as a slight testimonial of appreciation and affection. With the watch was a chain of filigree-twisted gold wire, connecting, at intervals of about six inches, topaz hearts set in a little rim of gold. It was desired that there should be one heart to represent each hospital, but no chain with exactly the right number of hearts could be found. Dr. McGee will probably value not less than the gift itself, letters sent with the various contributions which were bound together in leather, and accompanied the case containing the watch and chain. In acknowledging "the token of loving remembrance," Dr. McGee said: "This beautiful watch will be a daily reminder of the long days, full more of pleasure than of disappointment, which I have spent in the service of the army nurses, and its chain of linked hearts seems to bring a special message of affection from each of my dear friends, in our home parts in Cuba, in the Philippines, in China and Japan."

### A CANADIAN DEAN ON THE WORK OF NURSES.

Preaching in a London church on Sunday morning, the Dean of Fredericton, speaking of heroes, placed the work of devoted nurses on a level with that of soldiers who win the Victoria Cross, and went on to describe the unostentatious deeds of bravery of which the world hears nothing as important as that of the man who, in the words of the Book of Proverbs, "taketh a city." He specially mentioned a case which occurred on the other side of the Atlantic, during a severe epidemic of smallpox. A nurse, known as Sister Gloria, was in attendance on a patient, and notwithstanding all her care, the end had nearly come. Very thoughtlessly, and very reprehensibly, the poor soul, with failing breath, said to the sister, "Kiss me, nurse, before I die." Without a thought of herself, or of what the consequences might be, and only moved by the distress of the sufferer, she instantly complied. As Dean Partridge added, "God kept her from contracting the disease"; but, while admiring the self sacrificing spirit which prompted Sister Gloria to accede to a request which she felt was the last that would be made on earth, we think that she would have manifested greater moral courage, more regard for others, and for her own future usefulness, if she had kindly but firmly declined.

### LOOK AT THE LABEL.

In the course of an inquiry into the death of an old woman at King's Norton Union Infirmary the nurse in charge of the patient confessed that she had made a serious mistake. She took the carbolic acid bottle out of the cupboard where the methylated spirit was kept, and used the contents of it to rub the patient in order to prevent the occurrence of bed-sores. The immediate cause of death was diarrhœa, but the doctor added that he was not prepared to say that the accident might not have accelerated the end. The nurse pleaded that the bottles were of the same size, colour, and appearance; that the carbolic acid should have been in the poison cupboard, which was kept locked; and that it had been put in the other cupboard in error by another nurse. But when she admitted that she applied the contents without looking at the label, she cut the ground from under her feet. While we concur in the suggestion of the coroner that poison should be put into bottles easily distinguishable from other bottles, neither a similarity of appearance, nor an act of carelessness on the part of a colleague, is any excuse for ignoring the label. A nurse who omits to look at the label of any kind of mixture she administers is, in fact, guilty of a gross dereliction of duty, and we cannot doubt that the censure of the coroner in this case will teach others a lesson they will not forget.

### A SPLENDID RESULT.

The promoters of the bazaar at Dingwall on behalf of the District Nursing Association must be warmly congratulated upon the result of their efforts. The enterprise had the enthusiastic support of the leading people of the district, and from the opening—which was rendered specially interesting by a speech from Colonel Ross, who was wounded in South Africa and spoke eloquently of the work done there by the nursing sisters—to the close was carried on with remarkable energy. The stall-holders and their assistants were so irresistible, and the purchasers were so numerous, that at the close of the second day the handsome sum of nearly £600 was realised. This is exceedingly creditable both to the town of Dingwall and the county of Inverness, and affords the best proof that could be given of the value of the services rendered by the seven nurses attached to the District Association.

### ADDITIONS TO ANCOATS HOSPITAL.

As the result of the additions to the children's ward at Ancoats Hospital, Manchester, which have just been completed, nine more cots are now available. A suitable bath room and other offices have also been provided. Moreover, the nurses will in future have the advantage of possessing a large day room, which has been generously furnished by the committee; while six new single bedrooms for the use of the sisters, each room being supplied with hot and cold water, will materially augment the comfort of the staff, for whose convenience bath rooms of the most modern kind have been constructed.

### PITLOCHRY NURSING HOME.

The large attendance at the bazaar in aid of the Pitlochry Nursing Home shows that the affection with which Dr. W. S. Irvine, to whom the Home is a memorial, was regarded in life has not ceased because he has passed away. As Sir Alexander Muir Mackenzie, in performing the opening ceremony, said, the bazaar was intended to put the climax on the founding of the memorial "to one of the very best friends the people of Strathtay and Atholl ever knew." Its object was to provide the furniture and equipment for the nursing home, the site having been given, and the home itself having been built. Happily, the proceeds amounted to a sum sufficient for the purpose, and the memorial to Dr. Irvine has therefore been completed. A happier one, nor one more in accordance with his well-known sentiments, could not have been selected.

### A SAD RESULT OF CARELESSNESS.

Nurses do not as a rule need to be told to be careful about their patients, but many, we fear, are rather careless in running risks on their own account which they need not encounter. A pupil at the Hartford Hospital Training School for Nurses, in the State of Connecticut, named Hathaway, while nursing a baby with purulent ophthalmia, contracted the disease, and has since become totally blind. With due precautions there is but little danger, and the unfortunate young woman must have failed to fully estimate the risks she ran. We are very glad to see that a fund has been raised by the citizens of Hartford for her benefit, and every nurse on this side of the Atlantic, while feeling that her terrible experience supplies a fresh proof of the importance of individual care, will deeply sympathise with her in an affliction which has closed her career at the outset.

### INSUBORDINATION AT NEWTON ABBOT.

It seems impossible to preserve peace at the Newton Abbot Workhouse Infirmary. In the present case the guardians are not directly to blame; the culprit is one of the nurses who, according to the statement of the master, broke her leave on six occasions during a period of thirteen days in spite of his admonitions. It can be readily understood that her misconduct greatly interfered with the discipline of the house, and the visiting committee in deciding to reprimand and discharge her, took the very proper course. In aggravation of her offence, however, she left the workhouse without notice. We are afraid that the Newton Abbot guardians will not obtain model nurses until they have acquired a reputation for treating them with more consideration; but their mistakes cannot be accepted as a pretext for insubordination.

### SHORT ITEMS.

It is announced that the King has conferred the Royal Red Cross on Mrs. Lucy Scott and Mrs. Mary Davis, "in recognition of service to the sick and wounded in Natal."—The next examination of the Medico Psychological Association of Great Britain and Ireland will be held on Monday, November 4th, 1901. Schedules can be obtained from Dr. Benham, City Asylum, Fishponds, Bristol, to whom they should be returned before October 7th.—The Duke of Portland has promised an annual grant of £35 towards the expense of maintaining a qualified nurse for the service of the parishioners of Bolsover.—Nursing Sisters A. M. Glenie, A. Hill, and W. L. Harris are returning from South Africa in the *Dilwara* which is due at Southampton on the 15th.—The Queen's Jubilee Institute for Nurses has received the sum of £500 from the executors of the late Mr. Richard Bowerman West.—Sister Gift Kate Swanton, of the Army Nursing Service Reserve in South Africa, has been discharged from hospital to duty.

### Ophthalmia Neonatorum.

A Clinical Lecture by ROBERT JARDINE, M.D., etc., Senior Physician to the Glasgow Maternity Hospital.

TO-DAY I wish to draw your attention to an infant whose eyes are very badly inflamed. This is a case of what is known as ophthalmia neonatorum. At one time it was a perfect scourge in maternity hospitals, and blindness often resulted from it. You often hear the expression "blind from birth." A child may be born blind, but this is exceedingly rare. In a good many thousand births I have never seen a child born blind. The sight is usually all right at birth, but it may be destroyed a few days later if ophthalmia sets in, and proper treatment is not adopted. You can thus understand how serious a condition it is. By the adoption of preventative measures we have almost entirely banished it from our maternity hospitals, but even with the greatest precautions a case occasionally crops up. If you should have a case remember that the utmost care must be taken of it or blindness may result. I want to impress upon you how great your responsibility is in such a case. Remember that a little want of care may condemn an individual to go through life bereft of sight.

*The Cause of the Inflammation.*—I have no doubt you are all familiar with the usual old wives' explanation, viz., "a waft of cold in the eyes." You may at once disabuse your mind of this explanation. Cold has had nothing to do with it. The true explanation is that the eyes have been infected by vaginal discharge during the passage of the head through the birth canal. In all cases arising within three days of birth, if they are at all bad, a micro-organism can be found in the pus, and it is the essential cause. It is the organism which causes gonorrhœa. You may think that if the woman has gonorrhœa the disease could be easily detected from the presence of a nasty vaginal discharge. As a matter of fact it is very difficult to diagnose gonorrhœa in a case of labour unless it is an acute attack. The organisms may be present in the vagina and nothing be evident to the naked eye to indicate their presence.

The mother of this child shows no evidence of gonorrhœa, and yet the eyes were undoubtedly affected during birth. The inflammation did not begin until the third day. In many cases it comes on much sooner, within a few hours of birth, and we have had one or two cases in which it was discovered immediately after birth. The eyelids become red and swollen, and there is copious secretion which very quickly turns to pus. The lids often get glued together, and when they are separated pus gushes out. The lining of the inside of the eyelids and the superficial layer of the eye—*i.e.*, the conjunctiva, is very much inflamed. If prompt treatment is not adopted the inflammation soon spreads deeper into the eye and ulceration of the cornea occurs. The eye may be completely destroyed. One eye only may be affected, but it is difficult to prevent it spreading to the other. More usually both are attacked at once.

*Treatment.*—Prevention is always better than cure, and this is decidedly true in these cases. If there is any vaginal discharge of a suspicious nature, a lysol douche should be given early in labour. In every labour as soon as the child's head is born the eyes should be carefully wiped. Boracic lotion may be used, or warm water does very well, especially in private practice. In private work all beyond this that is usually necessary is to carefully wash the eyes when the child is being bathed; but if there is any suspicion of gonorrhœa, a few drops of a silver nitrate solution (2 grains to the ounce) should be dropped into the eyes. Another form of silver solution which is now much used is protargol (10 to 20 grains to the ounce). The excess of the silver solution should be washed away with a weak salt solution. The salt turns the silver solution white by changing it into chloride of silver, which is insoluble. There is one thing I wish you to bear in mind—viz., never to use a perchloride of mercury solution along with a silver one. If you do a white opaque deposit will form on the front of the eye. In hospital cases the silver solution should be applied in every case. We use protargol 10 grains to the ounce. Beyond keeping the eyes clean nothing more is required in the vast majority of cases, but if there is any indication of inflammation commencing active measures should at once be adopted. If the case is entirely under your charge, don't take the responsibility of treating it, but get a doctor to see it.

The treatment we adopt is to thoroughly cleanse the eye with boracic lotion and apply protargol (20 grains to the ounce) twice or three times a day. In the intervals between the applications the eyes must be bathed very frequently with boracic lotion. If only one eye is affected the sound eye should have the protargol solution applied once at least, and the utmost care must be taken to prevent any of the discharge getting into it. To cleanse the bad eye small pledgets of absorbent wool should be used and then burnt. On no account should the sound eye be touched with anything which has been used for the bad eye. To thoroughly cleanse the eye or apply the silver solution the lids must be everted or turned out. You must be very careful to thoroughly sterilise your hands both before and after dealing with the case. Everything used for the baby must be kept for it alone, and must be thoroughly sterilised before being used again. The disease is so contagious that one case may affect all the other children in the hospital.

The strong silver solution will have to be applied until the formation of pus stops. Ice compresses applied over the eyes are very beneficial. In a few days the active inflammation usually subsides, and the child will be able to open its eyes, but the treatment must be continued until the eyes are perfectly well. A sulphate of zinc solution, 2 grs. to the ounce, used with equal parts of warm water, makes a very good application after the pus has stopped forming. It is more astringent than the boracic lotion.

I trust that you will bear in mind the responsibility of dealing with such a case. With care these cases do well, but without care blindness is almost sure to result. Remember also that you are dealing with a very contagious disease, which you may easily convey to your own eyes or the eyes of others.

### To Nurses.

WE invite contributions from any of our readers, and shall be glad to pay for "Notes on News from the Nursing World," or for articles describing nursing experiences, or dealing with any nursing question from an original point of view. The minimum payment for contributions is 5s., but we welcome interesting contributions of a column, or a page, in length. It may be added that notices of entertainments, presentations, and deaths are not paid for, but, of course, we are always glad to receive them. All rejected manuscripts are returned in due course, and all payments for manuscripts used are made as early as possible after the beginning of each quarter.

### A Fashion Producing Disease.

By Viscountess Harberton.

### An Unpleasant Truth.

An enormous amount of attention is bestowed at present on the prevention of diseases being spread by the diffusion of germs and microbes. But so far the general public do not appear to realise that short of direct intercourse with the sick, no better means for conveying infection could be devised than the petticoated style of dress universally worn by women. Here is one example of which I was an eye-witness. One day in the spring of 1900 some person had evidently been very sick on the pavement near Albert Gate, Knightsbridge. This place is crowded owing to omnibuses stopping, and I had the curiosity to stand and watch the passers-by for a few minutes. Some women did not notice the dirt on the pavement at all, and walked over the place trailing their dresses. Others clutched at their skirts, but either the back or one side of their dress dipped into it all the same. A few got the dress safely up, but the long underskirt trailed into it, so that when the dress was let down it also would come in contact with the abomination collected by the under petticoat. And only about one woman out of every five successfully avoided it. Now, if this happens in daylight and with something so visibly disgusting, what must be the amount of nastiness which women collect on their clothes in the dark and from less prominent objects

EXAMPLES OF RATIONAL DRESS.

### Infant Victims.

The people who take young children out to walk more especially collect filth of all sorts on their skirts, as, whether carrying a baby, pushing a perambulator, or leading a child by the hand, it is impossible for them to hold their dresses off the ground. Indeed, they rarely attempt this impossible feat! And the dress is seldom brushed on coming in but is trailed about all over the carpets where the children have their toys, and crawl about with their faces close to the ground, thus unavoidably inhaling all the dirty particles as they dry. In fact, it seems probable that some outbreaks of diphtheria, of which the source of infection has seemed undiscoverable, have most likely been caused in this manner; as people suffering from mild forms of the complaint are not too ill to go about as usual, and like other throat diseases it is likely to induce spitting. Boots are always scraped and wiped at the door, but skirts, which from their absorbent material are infinitely dirtier, are left till next day, and then as often as not brushed on or close to a bed, or else in a kitchen or pantry where food is likely to be contaminated by the dust brushed off.

### Unavoidable Results.

This horrible kind of clothing (for horrible it is when viewed in the light of reality) also lies on the floor when the wearer sits down, and therefore in trains and omnibuses, and sitting at railway stations, as well as going down steps, it positively soaks up whatever dirt there may be on the ground, and no so-called "short" skirt would be any better in this respect. And when we furthermore remember that being sick on the pavement is probably the result of illness of some sort, and that people suffering from bronchitis, consumption, and other lung affections are almost compelled to spit, there can hardly be any more certain way of diffusing diseases than by trailing and soaking our clothes in these repulsive things.

### Reformers' Hardships.

The costumes shown in the accompanying picture are so arranged that neither in walking nor in sitting can they come in contact with the ground. And though these particular figures have a cycle beside them the dresses are not intended exclusively for cycling. On the contrary, they are completely suitable for general everyday wear, and there are a considerable number of people anxious to wear some such dresses, but they are hampered by the attitude of the mass of the public who do not understand the danger and real unpleasantness of ordinary skirts. Employers, for instance, would be likely to discharge any woman coming to their shop or office except in the regulation trailing skirt. Clubs of all sorts, even those for tennis, croquet, etc., would be almost certain to refuse to allow anyone to enter except in skirted attire, and would decide that dirt and disease were preferable to the smallest innovation in the matter of dress.

A Form of Persecution.

For it is quite a mistake to suppose that the spirit of persecution is dead. It may be in a state of suspension in religious matters, but in social customs it is still rampant. And compelling women, wherever there is power to do so, to wear a dress at once awkward and injurious, and one that to those who understand its evils is simply revolting, is entirely opposed to the principles of liberty of which the English profess to be so proud.

Liberty set at Naught.

Singing "Rule Britannia" is evidence of nothing but a sort of excitement; but recognising the rights of others, even when they happen to be in a minority, to do as they please in matters which concern themselves only, is the true meaning of the word liberty, and to judge by what we see, it is far from being either understood or practised by almost any class of the people.

Nurses' Uniforms.

It is a frequent source of wonder to many, who can have invented the special dress worn by professional nurses, as it combines nearly every bad and dirty characteristic of fashionable dress without such beauty as may sometimes be conceded to this last. It always trails on the ground, so that besides the likelihood of conveying infection about the house, such outdoor exercise as the nurse has time for is either made tiring by her having to keep hold of her skirt all the time, or if left alone, it collects dust and mud in the manner before mentioned. And an invalid's room is certainly not the place any more than a nursery where disease germs should be scattered freely, as those in a weak state of health are of all people the most likely to fall victims. The headgear with streamers attached, must be most uncomfortable, and necessitate a parasol being held up in summer; while the clumsy cloaks requiring to be held in place also by the hands, are most inconvenient.

An Urgent Need.

It is quite Chinesely illogical to go on as we are doing now; with institutions for curing diseases, and congresses meeting to discuss learnedly how they may be prevented, as was done by the recent Congress on Tuberculosis, and at the same time to continue to regard the wearing of skirts which strew every sort of disease germ broadcast in our houses, with complacent approval. Such a custom as this dress may well be placed among those customs referred to by Rudyard Kipling in his last poem as

"All the obese, unchallenged old things, that stifle and overlie us."

The simplest and most obvious reforms are not always the easiest to bring about, but it should not be taken for granted that because the evils of any particular thing have not always been understood, an alteration is any the less urgently needed. The general adoption of a suitable and cleanly dress for women is unquestionably one of the greatest needs of the day.

### Sick Nurses for Shipboard.

By a Voyaging Nurse.

(*Continued from page* 291.)

I have sometimes thought that the Colonial Up-Country Nursing Association might be induced to add another and a sea-going branch to the valuable work done by them in providing European and trained nurses for those who fall sick in our colonies. Many of the invalids who have been so carefully looked after during a serious illness by nurses belonging to this association are ordered home on the first available steamer, and during a long voyage are left to such rough and untrained care as they can obtain from the saloon and cabin stewards.

The malarial fever patients and convalescing enterics, so liable to relapse in the variations of temperature experienced while passing from the tropics to a temperate climate, the chronic dysenterics and liver cases peremptorily ordered a change of climate need some sort of trained care during a three to six weeks' sea voyage. Perhaps at starting none were so ill as to need the entire services of a nurse. And even though they were, at many foreign ports European nurses are so scarce there would certainly not be enough to go round.

The Training of Native Women.

Nobody with a technical knowledge of hospitals and nursing can fail to see that we are making a great mistake in not urging the native women of our Eastern colonies to train as nurses. The native woman of Ceylon, India, and China possesses most of the qualities essential to good nursing. But very little is done to induce them to enter hospitals and undergo a thorough course of training. Much prejudice and caste tradition needs to be overcome before they will adopt the nursing profession. But so much excellent nursing material should not be wasted for the lack of effort. There never can be enough European nurses in the East to meet the requirements of the European sick. Nursing in the tropics is too great a strain, unless a white woman be strong above the average. And the cost of a trained European nurse in many of our colonies puts her beyond the reach of all but the very well-to-do.

Sea-going Nurses.

Many invalids, returning from all parts of the East, are not able to afford the expense of a passage home for a trained nurse, even were such a treasure obtainable in the tropical station whence they come. Each would be willing to pay certain fees for the priceless privilege of a daily attendance of a ship's nurse, after the visiting plan which many middle-class and chronic patients now adopt on dry land. That is a very different matter from bearing the entire expense of a nurse's passage when perhaps half an hour daily covers the nursing necessities of the case. Very many nurses would be quite willing, if a steamship company granted free passage and quarters, to depend for salary upon the fees received for attending the sick and invalided on board.

On a voyage from China, India, or West Africa, with a saloon full of persons ordered home, a nurse might do extremely well in the matter of fees. There would be no difficulty whatsoever in obtaining sea-going nurses either at a small salary, or with the power to charge fixed nursing fees for services rendered to sick passengers. It would open up a charming opportunity to many nurses to do a little travelling and globe trotting, as it does to young doctors who ship as sea-going physicians before settling down to the prosaic humdrum of general practice.

A Professional Position Indispensable.

Indeed, a few certificated nurses, attracted by love of the sea and the chance for foreign travel, have even taken positions as stewardesses on passenger steamers. But the post of ship's nurse should be kept quite distinct from that of a stewardess. It should be a professional position filled only by certificated and highly qualified nurses. Nobody who has travelled on the invalid-laden steamers from the East can doubt the need of a nurse on board. Life and health would be saved, and the sick voyagers spared much suffering and discomfort under the kindly care of a skilful nurse. Conditions on board ship are very charming to the strong and healthy, but I can imagine no more forlorn state than that of invalids put on board a crowded passenger steamer as a last resource in the treatment of some serious tropical trouble. In the Straits Settlements and at the Indian ports of call some of these invalids come on board in a half-dying state, many of them quite alone and dependent in their critical condition, and during a long and weary voyage, on such attention as overworked stewards and stewardesses can give them. These sick persons are constantly landed at intermediate ports. Their symptoms have become too serious to allow of them remaining on board without the care of qualified nurses. Special wards are provided in some of the intermediate port hospitals for the reception of seriously sick passengers from the steamers touching there. But a week or a fortnight's voyage sometimes stands between a passenger and these hospital havens of refuge by the way.

A Common Criticism.

Moreover, it is a very tragic matter to many invalids on their way home to the good climate of Europe, and suffering perhaps from a disease which makes it imperative that they should be transferred quickly from the tropics, to be carried from their homeward bound steamer and be left behind in the wards of a tropical hospital. A common criticism of such cases is that "patients so ill should not be allowed to come on board without a nurse." Those who know the tropics and tropical diseases and conditions understand that there could not possibly be sufficient nurses to accompany each case, leaving the matter of cost entirely aside. Many such passengers who merely feel "seedy" when they embark, develop serious illness after a few days on board. Old fevers re-awaken in the system, grave liver symptoms re-assert themselves. Passing from the tropics to a colder temperature causes some passengers to develop dysentery and allied troubles, and many of these are perforce left halfway home at Colombo or Port Said. Or if they are able to continue their voyage they are unable to obtain under present steamship conditions the nursing and attention which such invalids so badly need.

The Present Conditions.

Berth and deck stewards and stewardesses are often most willing and ready to perform extra services and give special attention to a specially sick person. But their time is limited. Usually they are fully occupied with their normal duties, and their working hours are very long indeed. They cannot devote much time to the invalid, and they know nothing even of general nursing, still less can they be expected to understand the diseases of the tropics from which the shipboard invalids from the East are mostly suffering. A sick person confined to a cabin can count only on a very limited and crude form of care and attention. How such ship invalids would look forward to and value the stated visits of a cheery professional nurse to make afresh their bunk beds and to surround them with some degree of sick-room comfort amid the weary tossing and turmoil of a rough sea voyage! A woman invalid on board confined to her cabin does not have her bed made for days together unless she is able to do this for herself. Ship's beds are made by stewards. It is not the duty of a stewardess to make up bunks, nor has she time to do so. So that unless a woman patient can bring herself to the point of sitting up in a dressing-gown while her cabin steward makes her bed, it will remain unmade. Many "bad sailors" and delicate persons suffer most severely during a long voyage from the exhaustion consequent on prolonged sea-sickness. They would be only too glad to pay for a little attention from a trained nurse. The present method of caring for such sea-sick invalids is primitive and most unrefined. A ship's nurse would not be expected to attend to ordinary *mal de mer* patients; but in severe cases the sufferer has every right to be classed among ship-board invalids, and is entitled therefore to some degree of skilled nursing care.

*(To be continued.)*

### Entertainment to District Nurses.

A very pleasant gathering of members of the Notts Nursing Federation has just taken place at Sherwood Lodge. They were invited by Miss Seely, and district nurses attached to the Hucknall, Sutton, Kirkby, Wollaton, Balderton, Carlton-on-Trent, Beeston and other associations were present. In a thicket near the Lodge the Tibshelf Ambulance Band played, and the Nottingham Dolce Quartette party rendered a number of glees. Tea was served in the dining-room, and subsequently the party adjourned to the tennis courts, the bowling green, the conservatories, and the gardens, many of them promenading on the walks beneath the shade of some stately trees—relics of old Sherwood. The nurses were photographed, Miss Seely occupying the central position in the group. Both she and Sir Charles Seely were indefatigable in their efforts to render the visit enjoyable.

### Appointments.

Accident Hospital, Mountain Ash, N. Wales.—Miss Barbara Mackay has been appointed matron. She previously held the post of assistant matron at the Chelsea Infirmary, which she resigned in March last.

Ayr County Asylum, Glengall, N.B.—Miss Margaret Alison has been appointed matron. She was trained at the Longmore Hospital for Incurables, Edinburgh, and the Western Infirmary, Glasgow. She has since been assistant matron at the Stirling District Asylum, Larbert.

Chester-le-Street Union Infirmary. — Miss E. E. Willis has been appointed superintendent nurse. She was trained at Crumpsall Infirmary, Manchester, and has since been superintendent nurse at Easington.

South Eastern Fever Hospital.—Miss Annie Lee, Miss Agnes Annie Clark, and Miss Ellen Elsie Horner have been appointed charge nurses. Miss Lee was trained at the Poplar and Stepney Sick Asylum; Miss Clark at the Stanley Hospital, Liverpool; and Miss Horner at the Royal Infirmary, Newcastle-on-Tyne, and Tynemouth Infirmary, North Shields.

Swansea General and Eye Hospital.—Miss Mary Preston has been appointed assistant matron. She was trained at Liverpool Royal Infirmary, and has since been sister and temporary assistant matron at the Worcester General Infirmary.

Victoria Hospital, Frome.—Miss Mary Briggs has been appointed matron. She was trained at the Nursing Institute, Bangor, and has since been lady superintendent of the Nurses' Home at Frome for 15 years.

### Nursing in Irish Workhouses.

By Our Special Correspondent.

### CONFERENCE IN DUBLIN.

On Thursday last a conference of delegates from Poor Law Unions in Ireland was held in the board-room of the South Dublin Union Workhouse for the purpose of considering an order issued by the Irish Local Government Board on July 5th, dealing partly with the question of nursing, and to take counsel as to "the best course to adopt for the protection of the public interests." Delegates attended from 20 unions, the total number of unions in Ireland being about 160.

Mr. Mooney, Chairman of the South Dublin Union, was elected chairman. They were, of course, he said, aware of the purpose for which they were assembled. The meeting had been called in pursuance of a suggestion contained in a resolution passed by the Guardians of the Cashel Union on June 18th last. He had no particular knowledge about the matter himself, except that he knew that an order was issued by the Local Government Board some few months ago dealing with this nursing question. It was appealed against by the Armagh Board of Guardians and a great many other boards throughout the country, and they appeared by counsel before the Privy Council and gave evidence in opposition to it. That order appeared in a general way to take a great deal, if not all, the management of the workhouse hospitals out of the hands of the guardians and to place it in the hands of the Local Government Board. The Local Government Board were to be the judges of the number of nurses to be employed in workhouse hospitals; they were to prescribe the pay and qualifications; and they were to have control over their dismissal or retention. So that the Boards of Guardians, as he had said—and he gave evidence before the Privy Council—would simply be the paymasters of those officials. The Boards of Guardians resented this very strongly, with the result that the Lord Lieutenant and Privy Council refused to approve of the order, and it was withdrawn. A fresh order was issued on July 5th last, and, as far as he could judge without actually comparing the two—he had not time to go into the matter very closely—the latter modified the former to a very slight extent, and he considered retained most of its objectionable features. He was not prepared to suggest anything very definite with regard to the matter. Certain resolutions had been prepared which would be offered for their consideration, and he hoped that some course would be decided on which would have the effect of keeping the control of the matters in question in the hands of the Boards of Guardians who had been appointed by law to deal with them. There was such a demand for trained nurses and so few institutions were authorised to give them certificates that the salaries of trained nurses had gone up very high, and therefore something should be done towards providing a large supply of such nurses.

Mr. O'Neill, Magherafelt, moved the first resolution as follows:—

"That we consider the training monopoly enjoyed by a few hospitals is unreasonable in itself and unjust to the excluded hospitals and their officers and to the taxpayers generally, who have been mulcted thereby in salaries out of all proportion to the circumstances of the country and to the initial expenses of the recipients."

The resolution, he said, dealt with a most important aspect of this question. The nursing question had given great trouble and annoyance in the North of Ireland. In many cases it was almost impossible to obtain the services of trained nurses at any salary that could be considered reasonable. This resolution, he thought, should be adopted. In the Derry Infirmary the medical staff consisted of some of the most eminent medical men in the North of Ireland, and they were quite willing to train nurses, and had every opportunity of doing so, but because the beds in the infirmary were under the number required by the Local Government Board there was no power to train nurses. Yet they could be easily trained in a manner that would give every satisfaction and enable them to discharge their duties. If nurses could be trained in all the county infirmaries they would be able to obtain trained nurses at much smaller salaries than those they had to pay at present. The grievance of the Derry Infirmary had been brought under the notice of the House of Commons by Mr. W. Doherty, a member of the Irish Party, and the reply of the Local Government Board was that they could not consent to a reduction of the limit as to beds.

Mr. Corvery, Magherafelt, seconded the resolution. He contended that in the Magherafelt Hospital, in which the cost of nursing had in recent years enormously increased, there was no necessity whatever for a second trained nurse. They took on probationers under a trained nurse, but the Local Government Board would only recognise the latter. They had patients, about 31 being chronic cases not requiring a skilled nurse or doctor to attend to them. All that was required in the rural districts was one trained nurse, with the assistance of other ladies. All the old people wanted was to be attended to and kept clean, and given their meals. The new rules took out of the masters' hands the calling in of nurses, and that was most unfair. He did not intend to reflect on the medical officers, but it was only natural that they should appoint these nurses. (Several voices: "No, no.") Where there was a trained nurse for the Infirmary and the Fever Hospital, probationers were quite good enough for the work.

Mr. Kennedy, Rathdrum, said he assumed that the meaning of the resolution was that where they had a sufficient staff the union hospitals should be allowed to train. It was not too clear.

Mr. O'Neill said it was the intention of those who drew up the resolution to have it apply to the county infirmaries rather than the rural workhouse hospitals.

Mr. Kennedy said they should be in favour of such a hospital as this of South Dublin, for instance, that had a certain number of beds, being empowered to give certificates. If the resolution meant that paupers should be nursed by paupers he was opposed to it. In the case of religious orders of ladies, they should be recognised where they could show sufficient evidence of training, even where they had not gone through the regular training.

The Chairman said the resolution did not cover the whole case, and only protested against the monopoly held by certain hospitals to the injury of the taxpayers, a monopoly which was unjust to the excluded hospitals.

Dr. Laffan, Cashel, said it was not proposed for a moment to go back on the old system of nursing. It was not proposed to substitute untrained nurses for trained ones, but it was proposed that the small hospitals should have the same power to train, say, three or four nurses, as the big hospitals had to train forty or fifty. At present, salaries of from £50 to £60 a year had to be paid for trained nurses, owing to the monopoly held by a few hospitals in Dublin Where a hospital was able to satisfy competent opinion of its ability to train, it should be permitted to do so, and under such a system the training could be done more cheaply than at present. When pauper nursing was being done away with, he pointed out before the Workhouse Reform Association that the present monopoly would be created, and he suggested that there should be a competent central board composed of both laymen and medical men empowered to give certificates. There were about 100,000 persons treated in the workhouse hospitals of the country every year, while in the Dublin hospitals there were only a few thousand, and it was a singular anomaly that a few hospitals which contained only a fraction of the sick of the country should turn out the whole nursing staff. If the sick in the workhouses are different from the sick in the Dublin hospitals, why are they treated only by Dublin-made nurses? He did not propose that any hospital should be allowed to train without proper inspection. He knew of cases where hospitals proposed to train in three months for a handsome *douceur*; others proposed to train with the assistance of elaborate works of six and seven hundred pages in length, when a penny text book, such as that used in the English board schools, would be quite sufficient. And they had to pay through the nose for this monopoly. Under the new order of the Local Government Board the nurses will presently be costing up to £100 a year. One hospital in Dublin had a nurse to five beds. Why should not a rural hospital with 30 beds train several nurses? They had no guarantee at present that the certificate represented anything. There should be a uniform curriculum, drawn up by a body of competent laymen and medical men. The hospital applying to be recognised should prove that it was able to teach, and only a limited number of probationers should be allowed. As the certificates are given at present, each hospital has a school of its own. Where are the examinations held, and what is the nature of them? The English nurses approved of his views, and were anxious for a public examination and common curriculum. "I hereby certify that So-and-So has studied in Such-and-Such Hospital, Dublin," might mean nothing whatever. This was a resolution against returning to the old system, but they wanted a *bonâ fide* system of training and examination, which the present was not.

Mrs. EVERARD, Navan, said that in the Kensington Infirmary, they trained all their own probationers. They had a beautiful hospital there, and the matron told her they trained all their own nurses. If they were able to do that there, why not here? She did not see why ten beds should not be able to give as much instruction as two or three hundred. Hospitals that could prove they were able to train should be allowed to do so.

Mr. GREW, Lurgan, supported the resolution. He said they had in the infirmary there 200 patients on an average. They were up till recently allowed to train probationers who were passed by a committee of medical men in Belfast. The Local Government Board, however, would not now permit them to do that. They had experienced great difficulty in getting trained nurses and had to increase the salary offered several times. The Local Government Board had acted in a high-handed manner in the action they had taken, and he hoped the guardians would make a stiff fight and bring them to a sense of justice.

The resolution was carried unanimously.

Mr. BELL, Newry, moved the following resolution:—

"That we think there should be a uniform standard and curriculum of training for the various classes of nurses and a public examination at the end thereof, and that due provision should be made that this curriculum—which should be drawn up by a central committee—be faithfully adhered to."

Dr. HALL, Monaghan, in seconding the resolution, said it was absurd to say that nurses he had trained himself in the infirmary and the workhouse hospital, where they had to assist at all kinds of operations, who were quite competent, and indeed had been complimented by some of the Dublin surgeons, were not fit to take charge of the wards of a union hospital. He preferred the nurses trained by himself, and considered them better than strangers. But the Local Government Board insists on a nurse with a certificate. His board had to advertise twice for a certificated nurse, the supply was so limited.

The CHAIRMAN said it was a fact that nurses could get the certificates if they had sufficient money to pay. He understood that they could get through in a year for a certain sum, in two years for a smaller sum, while it took three if there was no money at all.

The resolution was passed unanimously.

Dr. LAFFAN moved:—

"That a committee be appointed, to be denominated the Association of the Unions of Ireland; that each union be invited to send a representative; that one-third of the members shall go out of office each year, and that the function of the said body shall be to look after the interests of the various boards, and to make representations from time to time on their behalf to the Local Government Board and to the public at large."

He said they should appoint a permanent body which would give effect to the decisions of that conference. If they broke up without leaving something permanent behind them, there might be nothing come of their proceedings but a report in the papers.

Mr. O'NEILL: Why not ask the General Council of the County Councils to act for us?

The CHAIRMAN: It is outside their duties.

Mr. KENNEDY seconded the resolution and it was passed unanimously.

Mr. SIMPSON, Armagh, moved:—

"That the framing of a curriculum for nurses, the preparation of a list of hospitals willing and able to carry out such curriculum, and of the securities to be taken for its enforcement, together with the preparation of a syllabus of examination and the arrangement with the Local Government Board for the reception of a deputation on the whole hospital question, be relegated to the committee."

The resolution was carried.

Mr. KENNEDY moved:—

"That this Conference is of opinion that the various members of religious orders who have had experience in nursing, and who may be appointed as nurses in workhouses, should be regarded as trained nurses by the Local Government Board."

The resolution having been seconded,

The CHAIRMAN said that those who had experience of the nursing of the Sisters of Mercy were agreed that it was excellent. Nurses belonging to religious orders with ten and twelve years' experience were not regarded as trained by the Local Government Board, although quite competent. This question of religions of the nurses had a good deal of interest for those who thought about economy. It was with great difficulty they got the Catholic sisters introduced in that union; but, after some experience, those who had opposed them agreed that they had been wrong. They also had Protestant sisters belonging to an English nursing order called deaconesses. The nuns and the deaconesses were paid £30 a year, with apartments, but no rations. When the deaconesses' order, from which they received a subvention, ceased to exist, they had then to be paid £60 a year, while the nuns still get only £30.

Mr. GREW supported the resolution.

Dr. LAFFAN said he knew nobody who could manage the sick so well as the nuns do. There was no conflict whatever between the trained nurse and the nuns in his hospital.

Mr. O'FARRELL said he knew plenty of people who would prefer, if sick, coming there to going into one of the regular Dublin hospitals, owing to the presence of the nuns. When they satisfy patients, doctors, and all concerned, why weren't they acknowledged?

The resolution was carried unanimously.

A vote of thanks to the Chairman concluded the proceedings.

### Echoes from the Outside World.

### AN OPEN LETTER TO A HOSPITAL NURSE.

THE late Empress Frederick has lived for many years so quietly and unostentatiously that the amount of money she has left must have come as a surprise to most people. Her English dowry and her accumulated savings, have totalled up altogether to half a million, some say even more; and this is notwithstanding that she declined to receive any legacy when Queen Victoria died, because she said she did not require any addition to her in come, whereas there were many members of the Royal Family who really stood in need of money. To each of her children the Empress Frederick bequeathed £50,000, including the German Emperor, and the Friedrichshof Castle at Cronberg has been apportioned to Princess Margaret, who married Prince Frederick Charles of Hesse, to whom an extra sum of money is also given that it may be properly kept up. It is not generally known how the castle came to be built. It was an investment from two legacies, one of which came to the Empress unexpected. The Duchess Galliera of Italy willed her £150,000, and a rich inhabitant of Berlin, Herr Tornow, left her another £50,000. The latter possessed a museum of arts and trades, in which he took a most enthusiastic interest, and during his lifetime the Empress often encouraged him to write about it, and liked to hear of his new additions. Out of gratitude he made the royal lady his heiress.

ALTHOUGH Princess Marie of Edinburgh married at such an early age that she was not widely known in England, English people have always shown a good deal of affection for her. She has now been a wife some nine years, although she is only about 25 years of age, and is still remarkably pretty. Lately she and her husband, the Crown Prince of Roumania, have been staying with their children at the royal castle of Pelesch, as the guests of the King and Queen of Roumania. The little ones adore their grandfather, and are always trying to find some little gift to present to him, a few flowers, a cheap basket or mat of Roumanian work, a childish drawing, or home-made piece of needlework; anything, in short, which will serve to show their love. One day the eldest, Prince Carol, who is eight years of age, during one of his walks in the nearest little town—the castle is situated at the foot of the Carpathian Mountains, and has most splendid views—saw in a shop a tiny statuette which he thought would please the King, so he inquired the price. "Fifty francs," was the reply. The boy had only twenty francs, but without hesitation he informed the shopman that he did not possess so much as fifty francs just then, but he would pay the twenty down and "owe him the rest." The vendor, recognising, of course, his royal little customer, willingly acquiesced. When the present was made the King was much pleased with the figure, and asked the boy how he obtained it. "I bought it," was the answer. "Where did you get the money, Carol?" "Oh, I had twenty francs which I paid the man, the rest I shall owe him." The King looked grave; but perhaps thinking that it was hardly fitting under the circumstances that he should reprimand the child himself, merely exclaimed, "That boy is a real Roumanian!" I cannot help hoping, however, that the matter reached the ears of his parents, and that they wisely seized the opportunity to tell the little chap that justice comes before generosity, and that those who cannot pay should wait till they can. That treasures can be purchased at will and paid for when convenient is a fatal belief for a royal prince of eight to hold, for to what extent will the idea have expanded by the time he is eighteen?

THE two excitements of the week—for the arrest of the Transvaal ex-official, Dr. Krause, for high treason is only a preliminary step—have been the Chinese comedy at Basle, and the coming of the Colorado beetle. Day by day, fresh details of the former have been forthcoming. Prince Chun, the brother of the Chinese Emperor, with a suite of fifty, was on his way to Berlin to apologise to the Emperor for the murder of the German ambassador during the Pekin riots. When he got as far as Basle he became seriously indisposed, too much so to proceed on his journey, because he had heard that he was expected to show the penitence of his country by personally "kow-towing" to the Kaiser, and by ordering his suite to completely prostrate themselves. This, according to some of the papers, he would never do, he would sooner die first; according to others, like a true patriot, he would go through with his penance and commit suicide immediately after. Now, after some days' residence in Switzerland, the German Emperor being seriously inconvenienced by the delay, it is announced that the dreadful formality is to be dispensed with, and regret will be expressed by the Prince in a perfectly simple way. Until the apology has been made the Chinese emissary will stay at the Orangery, at Potsdam, where ten special cooks have been engaged to do the necessary cooking. Afterwards the Prince will be free to go where he chooses. As to the unwelcome beetle, none of us would know it if we came across it, but it is said to be about less than half an inch long, of a yellow colour, with black lines down its wing cases, and reddish yellow and black legs. It breeds very rapidly, preferably feeds on potatoes, but will put up with tomatoes or poppies sometimes.

THE unsettled weather of the past week made one regret the less the proximity of the time for returning home. Not that Weymouth is at all a bad place in which to be weather-bound for a while. The Parade dries up so fast when the storm is over that one can get out at once, and even when the wind and rain are at their fiercest, there is always something of interest going on. One dull day, we took the train to Portland. The railway station is placed on the narrow neck of land which connects the island with the mainland. The weather was not sufficiently encouraging to make a pleasure of hiring a carriage and driving all round the place, so we just strolled on to Fortune's Well which is the capital of the island. There is nothing striking to be seen except the inn, which is proud of the fact that George III. visited it, and a fossilised tree affixed to the walls of one of the houses. Had we so desired, we could have gone to Verne Citadel, and perhaps have been sufficiently fortunate to see over it, but I do not feel much interest in impregnable forts, well mounted with guns, though possibly I might alter my ideas if Great Britain were threatened with invasion.

BY AND BYE we found ourselves on the top of a hill with a fine view over Chesil Beach, which is a long flat stretch without verdure of any kind. In fact, we came to the determination that Portland by its very surroundings seemed predestined to be the site for a prison and all the horrors and sadness attached to it. It was mid-day, and as we approached the prison itself we noticed a small crowd, the gates swung back, and gangs of convicts attended by warders and armed soldiers passed out on their way back to the stoneyards, where they prepare and dress the celebrated Portland stone. Lots of the spectators—evidently it is the correct thing to stand and watch the melancholy procession—laughed and made remarks as the men filed by. But it made me horribly inclined to cry. Some of the prisoners were so young and so "good" looking that it seemed difficult to understand their position, though, of course, a few were typical "gaol birds" and seemed to like being stared at. It was pleasant after lunch to stroll through the village of Easton with its quaint stone-roofed houses, till we found ourselves in the pretty Cove of Church Hope, the only part of the island where there are any trees. Pennsylvania Castle we did not go over. The next day saw us whirling back to London again, and we specially chose a train which came to Waterloo *viâ* Bournemouth, so as to enjoy a glimpse of the pine-clad heights and pleasant surroundings of the ever popular resort. Judging by the number of people at the Central Station crowds of visitors are staying there.

### Everybody's Opinion.

[Correspondence on all subjects is invited, but we cannot in any way be responsible for the opinions expressed by our correspondents. No communication can be entertained if the name and address of the correspondent is not given, as a guarantee of good faith but not necessarily for publication. or unless one side of the paper only is written on.]

### TRAILING SKIRTS.

"L. T." writes: In your article on this subject you say that no practical suggestion has been made except by Lady Harberton, but may I be allowed to say that both in the *Times* and in the *Queen* I have made one, which for the present at least, till the absurd and objectionable fashion is given up, will do away with its chief evils, and that is, to loop up the dress behind with buttons at the waist, to be used out of doors, if trains are desired in the house for ornament, or for the sweeping up of dust. This would entail no immediate change in the dresses, which can hardly be looked for at once.

### HINTS ON THE MANAGEMENT OF CONVALESCENT HOMES.

"O. C. E." writes: I was much interested in the article by the Matron of a Yorkshire Convalescent Home, but as regards herself and the assistant matron baking the bread, surely it would take a long time to make sufficient, and in that case who did their work? Our matron is much too busy a lady to be able to perform such duties, and I am certain that I could not possibly have time to do it. Also, one wonders if the patients do not leave as in other convalescent homes, as the Yorkshire matron says that her one lecture, and showing of wasted bacon, when she first went there, was sufficient to prevent waste ever since. Patients as a rule are only allowed to stay a few weeks in convalescent homes.

### A QUESTION OF INTEREST.

"Locum Tenens" writes: I agreed to take matron's locum work for three weeks (without salary), from September 10th. I made my arrangements to suit the matron's convenience, and declined other offers. On August 29th, I hear from the matron that she has altered her plans, and that my services will not be required. Would you kindly tell me if I am entitled to compensation for the expense I shall be put to for three weeks' board and residence, an expenditure entailed on me by the matron's failure to carry out her agreement. I ask in the interest of the nursing profession, as it may be useful for both sides to know their liabilities in making agreements of this kind.

[We are afraid that our correspondent, with whose disappointment it is impossible not to sympathise, has no legal remedy. Her misfortune is one that might befall any person voluntarily offering to undertake another's duty. From the letters which our correspondent encloses for our information, we gather that the breach of engagement on the part of the matron was brought about by circumstances over which she had no control, and which she evidently deeply regrets. Editor, The Hospital Nursing Mirror.]

### WORKHOUSE NURSING.

"Dr. F. R. Humphreys" writes from 27 Fellows Road, N.W.: There is an old proverb that "Outsiders see the most of the game," and on that ground I venture to trouble you with a letter on the subject of "Workhouse Nursing." I suppose that it will scarcely be denied that, theoretically at all events, trained nursing is the only form of nursing which should be found in any hospital, whether workhouse or general. Certainly to no general hospital would the public subscribe a penny if the nursing staff were not properly trained. Complete restoration of the sick patient to health at the earliest possible moment is the object of all nursing. The practical difficulty of finding trained nurses willing to exercise their profession in workhouse infirmaries is, I think the chief reason why every workhouse infirmary, or, at any rate, why the majority of these institutions are not fully at the present moment staffed with trained nurses. For some time past I have been closely studying the possibility of providing trained nursing throughout the Poor Law institutions, and have come to the conclusion that there is not sufficient opportunity for training a sufficient number of nurses in the present institutions. Only about 10 per cent. of the provincial infirmaries of the present time are of a sufficiently large size (two hundred beds as a minimum) to give the requisite experience to the probationers, though a far larger number train or try to train on a far smaller number. The remaining 90 per cent. of the workhouse infirmaries are in great part very small. Some 67 per cent. contain on an average less than 45 patients. Not only is it impossible to train nurses in these small places, but it is impossible to keep up a staff of trained nurses in them. This is a matter of common knowledge, and due partly to the monotony of the life, partly to the infirmaries being within the workhouse walls, partly to causes connected with the want of accurate knowledge, to put it mildly, on the part of Boards of Guardians as to the conditions under which trained nursing can alone be carried on. The provision of training schools in sufficient number and of sufficient size; complete separation of the sick part of the workhouse from the repressive part; the adoption of measures which will have been found to best retain well-trained nurses for a considerable period in general hospitals and in other parts of the Poor Law service must therefore be generally adopted if the throwing away of the ratepayers' money upon an inefficient and wasteful system is to cease. A long study of the relative position of workhouses as they lie scattered about England, and the distribution of the patients in each, has convinced me that the infirmaries may be reduced to between one-fourth and one-fifth of the present number without obliging the sick to be carried or the friends or guardians to have to travel an undue distance from their own unions. This reduction in numbers would cause a considerable increase in the number of infirmaries large enough to be utilised as training schools without being of unwieldy size, that is between 200 and 500 beds. It would almost entirely do away with the small infirmaries, the crucial difficulty. Economically, it would do away with a considerable number of nurses, and it would reduce very considerably the expense of maintaining so many different establishments as at present, at least so far as the sick part is concerned. The institutions would then be capable of supplying their own vacancies with their own probationers, and of having a large surplus of trained nurses for other purposes. The other purposes would be the supply of trained nurses to those smaller institutions, 100 to 200 beds, which were sufficiently modern and well managed to make it possible to properly nurse in them. Again, the acutely sick, who could only be removed to the nearest union, would be attended to by a trained nurse sent down from the surplus staff of the larger institution to which it sent its patients. There would be a few isolated, very small infirmaries who would also be dependent on these large institutions, and as the infirmaries found in Wales are, with two exceptions, too small to train, and too isolated to be abolished, they would also be dependent upon the surplus staff. This scheme is, therefore, a union of unions, but instead of them being isolated from one another they would be all united together by the aid of a central body, who would be charged with the duty of keeping up the standard, acting as intermediary, and, like a guard ship, retaining the names of those nurses on the books of the service while they were on leave, or, as a temporary measure, while they were on a central list to which the guardians could apply for nurses to fill vacancies. The saving in the cost of advertisements alone by such a list would be very large. Finally the attractions which will have to be held out, firstly, to suitable women to enter as probationers, and secondly, to trained nurses to remain in, or to enter, the service will have to be very much increased if the services of these women are to be obtained in reasonable numbers.

### For Reading to the Sick.

THE SACRED NAME.

Jesu is in my heart, His sacred name
Is deeply carvèd there: but th' other week
A great affliction broke the little frame
Even all to pieces; which I went to seek:
And first I found the corner where was J,
After where ES, and next where U was graved.
When I had got these parcels, instantly
I set me down to spell them, and perceived
That to my broken heart he was *I ease you*,
And to my whole is JESU.

*George Herbert.*

Some day all doubt and mystery will be made clear,
The threatening cloud which now we see will disappear.

Some day what seems a punishment or loss or pain,
Will prove to be God's blessing, sent for very gain.

Some day our weary feet will rest in sweet content,
And we shall know how we were blest in what was sent.

And, looking back with clearer eyes o'er life's short span,
Will see with wondering glad surprise God's perfect plan.

And, knowing that the path we went was God's own way,
Will understand His wise intent some day—some day!

*Anon.*

Communion with God is the great fact of life. All our forms of worship, all our ceremonies and symbols of religion, find their meaning here. . . . Religion is not an acceptance of a creed, or a burden of commandments, but a personal secret of the soul, to be attained each man for himself. It is the experience of the nearness of God, the mysterious contact with the Divine, and the consciousness that we stand in a special individual relationship with Him. . . . Men of all ages have known this close relationship. . . . To us, in our place in history, communion with God comes through Jesus Christ. It is an ineffable mystery, but it is still a fact of experience. Only through Jesus do we know God, His interest in us, His desire for us, His purpose with us. He not only shows us in His own example the blessedness of a life in fellowship with the Father, but He makes it possible for us. United to Jesus, we know ourselves united to God.

*Hugh Black—"Friendship."*

Whoever can think of religion as an addition to life, however glorious—a starry crown, say, set upon the head of humanity—is not yet the least in the kingdom of heaven. Whoever thinks of life as a something that could be without religion, is in deathly ignorance of both. Life and religion are one, or neither is anything: I will not say neither is growing to be anything. Religion is no way of life, no show of life, no observance of any sort. It is neither the food nor the medicine of being. It is life essential. . . . A man to whom virtue is but the ornament of character, something over and above, not essential to it, is not yet a man.—*Geo. Macdonald.*

We believe that every man ought to be a Temple of the Living God. The life of a soul is sacred in every stage of its existence.—*Mazzini.*

### Notes and Queries.

**The Editor is always willing to answer in this column, without any fee, all reasonable questions, as soon as possible.**
**But the following rules must be carefully observed:—**
**1. Every communication must be accompanied by the name and address of the writer.**
**2. The question must always bear upon nursing, directly or indirectly.**
**If an answer is required by letter a fee of half-a-crown must be enclosed with the note containing the inquiry.**

*Probationers.*

(212) 1. Will you tell me what age a young person must be to enter as a probationer into a hospital? 2. Are wages paid from the first? 3. Is uniform found? 4. Would she be bound for any definite time?—*E. B.*

1. From 23 to 30. 2. In some institutions. 3. Generally. 4. Yes. See "The Nursing Profession: How and Where to Train" for full particulars.

Will you kindly give me particulars as to the best hospitals at which to train, and what salaries are attainable for a young lady of 20?—*M. D.*

Probationers of 20 are only eligible for some children's hospitals. See reply to *E. B.*

*Secretary and Dispenser.*

(213) Would you kindly advise me how I can get a post as secretary and dispenser at a small hospital or charitable institution? I have advertised in The Hospital, and other papers without success. Could you suggest any agency, or would you advise me to advertise again?—*Sphinx.*

The best way to succeed by advertising is to repeat an advertisement regularly for some time in the same paper.

*Sanitary Inspector.*

(214) Will you please tell me where I can receive instruction in Birmingham to qualify me as sanitary inspector? Can you give me any idea as to the cost?—*J. B.*

You might obtain the information you require privately from the Birmingham and Midland Institute; but Birmingham has no society for the instruction of sanitary inspectors.

*Private Home.*

(215) Could you, or any of your readers, recommend to the writer a good opening for a nurses' co-operation and private convalescent home?—*M. B.*

This is a subject on which it is impossible to advise.

Would you kindly tell me if there is an opening for a first-class nursing-home in Harley Street, or in that neighbourhood?—*M. S.*

See reply to M. B.

*Short Training.*

(216) Would you kindly advise me as to any hospital which would take lady probationers for short general training? I wish to acquire sufficient knowledge of nursing to fit me for taking a situation as efficient companion to some invalid not actually requiring a nurse.—*E. J.*

Several hospitals afford the training you seek. Full particulars of which are given in the "Nursing Profession: How and Where to Train." Possibly the Middlesex Hospital, Mortimer Street, W., would suit you as well as any.

Would you kindly tell me where I could get a year's general training, with uniform and small salary: I cannot afford to pay a premium?—*E. B. F.*

See the "Nursing Profession: How and Where to Train." You will not get a salary with only one year's training at a good school.

*L.O.S. Qualifications.*

(217) Will you kindly tell me at how many midwifery cases I should have to be present before going in for the L.O.S. Examination? 2. Are cases seen in the district sufficient? 3. What text-books are recommended? 4. Are there any correspondence classes in connection with the society?—*Nurse M.* (Dundee.)

1. You must give proof of having attended not less than twenty labours. 2. Yes. 3. "A Practical Handbook of Midwifery," by Francis W. N. Haultain, M.D., is much recommended. 4. The Midwives' Society, 12 Buckingham Street, Strand, W.C., would provide you with courses of instruction; and you might possibly get your local medical man to prepare you for the examination. Write to the Secretary L.O.S., 20 Hanover Square, for particulars of examination.

*Demand for Nurses.*

(218) Having seen in The Hospital Mirror that there is plenty of work for nurses in Cape Colony, I should be much obliged if you will send me full particulars as soon as possible. I am a private nurse.—*E. M.*

You might write to the Lady Superintendent, the Victoria Nurses' Institute, Capetown, Cape Colony. Can you afford to pay your passage and to keep yourself until you get work? If not, it is a great risk to go unless you have friends there.

*South Africa.*

(219) I am a three years' trained nurse, and have been four years abroad. I can stand any amount of heat and wish to go abroad again. I would like to go to South Africa and help to nurse the wounded, but how am I to get there? Can you advise me?—*E. M. K.*

Write to the Hon. Secretary, The Army Nursing Service Reserve, 18 Victoria Street, Westminster, S.W.; and to the Secretary, The Colonial Nursing Association, Imperial Institute, S.W.

**Standard Books of Reference.**

**"The Nursing Profession: How and Where to Train." 2s. net; post free 2s. 4d.**

**"Burdett's Official Nursing Directory." 3s. net.; post free, 3s. 4d.**

**"Burdett's Hospitals and Charities." 5s.**

**"The Nurses' Dictionary of Medical Terms." 2s.**

**"Burdett's Series of Nursing Text-Books." 1s. each.**

**"A Handbook for Nurses." (Illustrated.) 5s.**

**"Nursing: Its Theory and Practice." New Edition. 3s. 6d.**

**"Helps in Sickness and to Health." Fifteenth Thousand. 5s.**

**"The Physiological Feeding of Infants." 1s.**

**"The Physiological Nursery Chart." 1s.; post free, 1s. 3d.**

**"Hospital Expenditure: The Commissariat." 2s. 6d.**

**All these are published by The Scientific Press, Ltd., and may be obtained through any bookseller or direct from the publishers, 28 and 29 Southampton Street, London, W.C.**

## Figures and Tables

**Figure f1:**